# Whole Genome Sequencing for Studying *Bacillus anthracis* from an Outbreak in the Abruzzo Region of Italy

**DOI:** 10.3390/microorganisms8010087

**Published:** 2020-01-08

**Authors:** Alexandra Chiaverini, Mostafa Y. Abdel-Glil, Jörg Linde, Domenico Galante, Valeria Rondinone, Antonio Fasanella, Cesare Cammà, Nicola D’Alterio, Giuliano Garofolo, Herbert Tomaso

**Affiliations:** 1 National Reference Center for Whole Genome Sequencing of Microbial Pathogens: Database and Bioinformatic Analysis, Istituto Zooprofilattico Sperimentale dell’Abruzzo e del Molise “G. Caporale”, 64100 Teramo, Italy; c.camma@izs.it (C.C.); n.dalterio@izs.it (N.D.); g.garofolo@izs.it (G.G.); 2 Institute for Bacterial Infections and Zoonoses, Friedrich-Loeffler-Institut, 07743 Jena, Germany; Mostafa.AbdelGlil@fli.de (M.Y.A.-G.); Joerg.Linde@fli.de (J.L.); Herbert.Tomaso@fli.de (H.T.); 3 Anthrax Reference Institute of Italy, Istituto Zooprofilattico Sperimentale della Puglia e della Basilicata, 71121 Foggia, Italy; domenico.galante@izspb.it (D.G.); valeria.rondinone@izspb.it (V.R.); antonio.fasanella@izspb.it (A.F.)

**Keywords:** *B. anthracis*, outbreak, WGS, canSNPs

## Abstract

Anthrax is a serious infectious disease caused by the gram-positive and spore-forming bacterium *Bacillus anthracis*. In Italy, anthrax is an endemic disease with sporadic cases each year and few outbreaks, especially in Southern Italy. However, new foci have been discovered in zones without previous history of anthrax. During summer 2016, an outbreak of anthrax caused the death of four goats in the Abruzzo region, where the disease had not been reported before. In order to investigate the outbreak, we sequenced one strain and compared it to 19 Italian *B. anthracis* genomes. Furthermore, we downloaded 71 whole genome sequences representing the global distribution of canonical SNP lineages and used them to verify the phylogenetic positioning. To this end, we analyzed and compared the genome sequences using canonical SNPs and the whole genome SNP-based analysis. Our results demonstrate that the outbreak strain belonged to the Trans-Eurasian (TEA) group A.Br.011/009, which is the predominant clade in Central-Southern Italy. In conclusion, the high genomic relatedness of the Italian TEA strains suggests their evolution from a common ancestor, while the spread is supposedly driven by trade as well as human and transhumance activities. Here, we demonstrated the capabilities of whole genome sequencing (WGS), which can be used as a tool for outbreak analyses and surveillance activities.

## 1. Introduction

Anthrax is a serious infectious disease caused by the gram-positive and spore-forming bacterium *Bacillus anthracis*, which is able to produce extremely resistant spores that can survive in the environment for several decades [1]. Anthrax is a dangerous disease of animals known from ancient times to be also a major concern to humans.

The last global report found the disease still widespread, being most prevalent in developing countries and well-controlled within more economically developed countries [2,3].

In Italy, animal anthrax is an endemic disease with sporadic episodes occurring usually during hot dry summers and after a rainy springs, but it can be linked to human activities that interfere with soil surfaces [4]. In Southern Italy, few severe epidemic outbreaks have been recorded so far, involving a large area between Calabria and Basilicata regions, probably due to the actions of hematophagous flies [5,6,7].

In the last few decades, Italian anthrax epidemiology has become clearer, and much more is known about the endemic territories, but also new contaminated areas have been recently discovered.

*B. anthracis* is one of the most genetically homogeneous bacteria. The genotyping methods currently in use are: (i) the analysis of 13 canonical SNPs (single-nucleotide polymorphisms) (canSNPs) that are the most stable and not homoplastic loci useful for phylogenetic investigations and (ii) the multi-locus variable number of tandem repeats analysis (MLVA) that has higher resolution suitable for outbreak investigations.

Using the canSNPs, *B. anthracis* can be divided into three major lineages: A, B, and C [8,9]. The major group A is dispersed throughout the world, while group B is restricted to Africa (B.Br 001/002 and B.Br. Kruger), Europe (B.Br. CNEVA), and Russia [10]. Group C is a rare genetic lineage found in North America, but the real origin is unknown [8]. Supplementary subdivision could be achieved by using different MLVA panels [8,11].

Group A is subdivided into several sub-groups and sub-lineages scattered throughout the world with specific geographic distribution [12,13]. The A.Br. Vollum was found to be dominant in Southern Africa, the A.Br. Aust94 is prevalent in Western and Southern Asia and Western China, the A.Br.001/002 was found in Central and Eastern China, the A.Br.WNA in North America, the A.Br.003/004 in South America, the A.Br.005/006 was found in Australia, Africa, and Europe. Finally the sub-group A.Br.008/009, also known as the Trans-Eurasian (TEA) sub-group, is widespread throughout Europe [14,15] and Russia [16]. It occurs in Southern and Eastern Europe and represents the dominant sub-group in Italy [15]. The TEA splits into two sub-groups A.Br.008/011 and A.Br.011/009, adding a supplemental canSNP [9,17].

Although canSNPs are useful for resolving the major branches of the *B. anthracis* population and MLVA has more discriminatory power, these methods are not the most appropriate tools for *B. anthracis* genotyping [12]. Meanwhile, whole genome sequencing (WGS) has now emerged as a powerful alternative method, which can support both outbreak investigations and routine surveillance of *B. anthracis*.

Here we described the WGS analyses from an anthrax outbreak that occurred in Abruzzo. In order to investigate the origins of the outbreak, we compared the strains against 19 Italian genomes publicly available [12]. Furthermore, we downloaded 71 sequences of various *B. anthracis* strains belonging to different canonical SNP lineages that represent the global distribution of *B. anthracis* and used them to verify the phylogenetic positioning. To this end, we analyzed and compared the genome sequences using canonical SNPs and whole genome SNP-based analysis.

## 2. Materials and Methods

### 2.1. Outbreak Scenario

During summer 2016, an anthrax outbreak claimed the lives of four goats in a farm located at Prezza in the Italian region of Abruzzo. This region had neither reported anthrax in the last 50 years nor did any memories about the disease exist among the farmers from the affected districts.

One strain (2016.AZ.3512.1.7) from the goats was isolated and genotyped for better characterization of the outbreak.

### 2.2. DNA, CanSNPs, and WGS

DNA was extracted from the *B. anthracis* cultivated onto blood agar media using the Maxwell^®^ 16 Cell DNA Purification Kit with the Maxwell^®^ 16 Instrument for automated purification of genomic DNA. Total genomic DNA was quantified with the Qubit fluorometer (QubitTM DNA HS Assay, Life Technologies, Thermo Fisher Scientific Inc., Eugene, OR, USA) Sequencing libraries were prepared using Nextera XT library preparation kit (Illumina Inc., San Diego, CA, USA) according to the manufacturer’s instructions. The libraries were sequenced using the Illumina NextSeq 500 platform, producing 150 bp paired-end reads.

CanSNP profiles were obtained using 13 allelic discrimination assays involving specific oligonucleotides and probes, as described by Van Ert et al. [8]. Each 10 μL reaction mixture contained 1× TaqMan Genotyping Master Mix (Applied Biosystems, Foster City, CA, USA), 250 nM probe, 600 nM each of the forward and reverse primer, and approximately 10 ng template DNA. The thermal cycling parameters used were as follows: 10 min at 95 °C, followed by 40 cycles of 15 s at 95 °C and 1 min at 60 °C. Endpoint fluorescent data were acquired by using the AB1 7900HT instrument. The CanSNP data were compared with the data for 12 recognized sub-lineages or sub-groups.

The 14th SNP was detected using a high-resolution melting assay for a specific A.Br.011 CanSNP [9,18]. Amplification was performed using the CFX Connect Real-Time System (BIORAD, Hercules, CA, USA) and Precision Melt Supermix (BIORAD). The reaction mixture contained 0.2 μM of each primer and 1× Precision Melt Supermix (BIORAD) in a 20 μL final volume. The following cycling parameters were used: 2 min at 95 °C, followed by 35 cycles of 10 s at 95 °C and 30 s at 60 °C. The samples were then heated to 95 °C for 30 s, cooled down to 60 °C over 1 min, and then heated from 65 °C to 95 °C at a rate of 0.5 °C/s. High-resolution melting data were analyzed by using Precision Melt Analysis Software.

In order to compare the Abruzzo outbreak strain to the Italian strains, we downloaded raw paired-end sequencing datasets from 19 available strains. In addition, we downloaded 71 diverse paired-end sequencing datasets from the Sequence Read Archive (SRA) and used them for worldwide phylogenetic positioning.

As part of the download, we extracted epidemiological data and canonical SNP clades from the dataset published by Sahl et al. [12]. Sequencing IDs and epidemiological information for each strain are reported in Table S1.

### 2.3. NGS Data Analysis

For analyzing all paired-end sequencing data, we used our in-house pipeline, which is available upon request at GitLab [19]. For scalability and fast run-time, the pipeline is implemented within the Snakemake workflow management system [20]. Bioconda was used for package management and easy installation [21].

The pipeline includes steps for quality control of all samples using FASTQC [22], calculation of theoretical genome coverage, and taxonomic classification of the reads using Kraken [23].

Genome assembly of Illumina paired-end reads was performed using shovill [24] which uses SPAdes (v. 3.12.0) [25] as genome assembler with the option (-only-assembler). Based on the genome assemblies, the pipeline predicts virulence factors utilizing ABRicate (v. 0.8.13) [26] against the Virulence Factor Database [27]. To identify gene coding for antimicrobial resistance, ABRicate was used against the NCBI database (PRJNA313047) with BLAST thresholds set at 80% coverage and 75% identity.

Genotyping of *B. anthracis* was performed using whole genome alignment-based SNP analysis with Parsnp (v. 1.2) [28] and reference genome *B. anthracis* Ames Ancestor genome (GCA_000008445.1_ASM844v1) to guide the genome alignments. The alignment output obtained by Parsnp was used to construct a phylogeny of the outbreak strain in comparison to strains collected from different countries. Results were compared to the traditional canSNP typing method.

Additionally, we implemented a mapping-based whole genome SNP analysis using Snippy (v. 4.3.3) [29] and *B. anthracis* Ames Ancestor as reference genome (GCA_000008445.1_ASM844v1) to calculate a phylogeny for the outbreak strain in comparison to the 19 strains from Italy. We used FigTree v. 1.4.3 [30] and Bionumerics [31] for visualization of phylogenetic trees together with epidemiological data.

The generated reads from the strain (2016.AZ.3512.1.7) were submitted to the National Center for Biotechnology Information (NCBI) under the bio-project accession number PRJNA587863.

## 3. Results

### 3.1. Genomic Features of the Abruzzo Outbreak Strain

Genotyping results show that the study strain belongs to canonical SNP sub-group A.Br.011/009. Whole genome sequencing of the strain resulted in a total of 4,564,206 reads (615,149,873 nucleotides) with a raw average read length of 134 bp (35–151 bp) and an average coverage of 109x (Table 1). Genome assembly using SPAdes generated a draft genome that was composed of 35 contigs of length >500 bp with an N50 contig length of 514,228 bp. The total assembly size of the sequenced strain was 5,450,929 bp representing 99.03% of the reference Ames Ancestor genome (GCA_000008445.1_ASM844v1).

In total, we identified 13 virulence genes in the analyzed strain (Table 1). Moreover, eight antimicrobial resistance genes that confer resistance against fosfomycin, beta-lactam antibiotics, macrolide, glycopeptide, and vancomycin were identified (Table 1).

Sequence data for the publicly available *B. anthracis* from Italy (*n* = 19) included the raw sequence reads of 16 strains and the WGS genome assemblies of three strains.

The two virulence plasmids pXO1 and pXO2 were identified via the BLAST detection of the plasmid gene markers [32]. We detected the two anthrax plasmids (pXO1 and pXO2) in all strains analyzed from Italy except one strain (A0878), which was found to lack both plasmids (Table S2).

### 3.2. Worldwide Phylogenetic Positioning of the Abruzzo Outbreak Strain

The genomic sequence of the outbreak isolate (study strain) and 90 complete *B. anthracis* genomes (19 Italian sequences and 71 sequences previously published by Sahl et al. [12]) were used to construct a global phylogeny of *B. anthracis* based on the SNP analysis of the complete genomes (Table S1).

**Figure 1:**
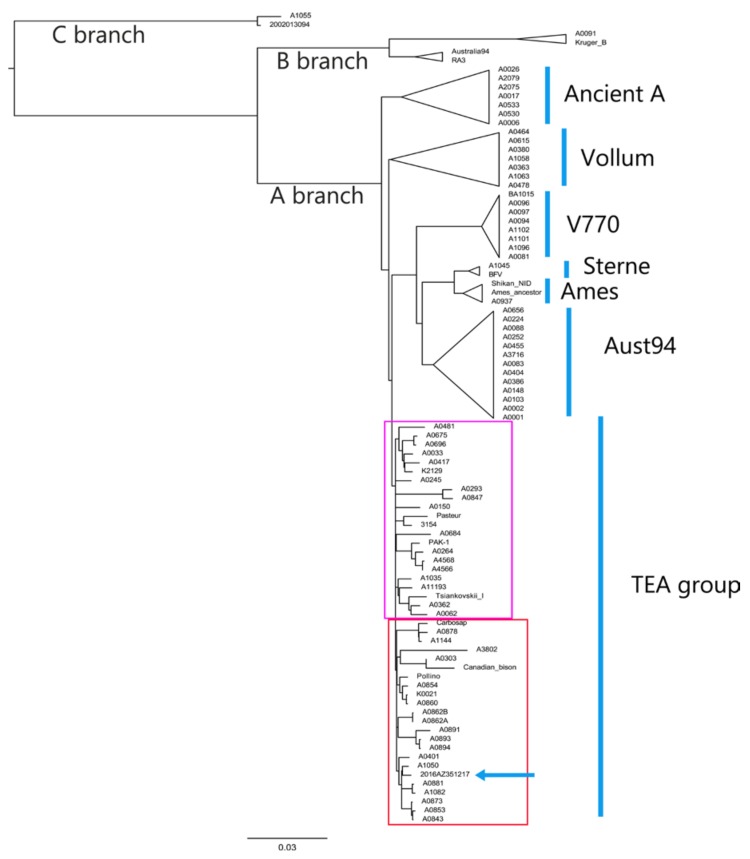
Global phylogeny of all 91 *B. anthracis* strains using Parsnp analysis output. In the red box are the strains belonging to the canSNP sub-group A.Br.011/009 and those belonging to canSNP sub-group A.Br.009 (Canadian bison and A0303). The blue arrow indicates the position of outbreak strain. The violet box shows the *B. anthracis* strains belonging to the sub-group A.Br.008/011. The study strain (2016.AZ.3512.1.7) is represented as 2016AZ351217.

The global phylogeny is reported in Figure 1, in which the information relating to the canSNP group, the country of origin, the host, and the year in which each strain was isolated are reported.

As reported by Sahl et al. [12], there were three major lineages where group A was the most observed around the world. In group A belonged A.Br.001 and 001/002, A.Br.002/003, A.Br.003/004, A.Br.005/006, A.Br.007, A.Br.009, A.Br. Aust94, TEA group, and A.Br. Ames, which is also called “Ancient A”.

Italian strains were assigned into two canSNP sub-groups: A.Br.008/011 and A.Br.011/009. The studied outbreak strain belonged to the Trans-Eurasian (TEA) sub-group A.Br.011/009 together with all 17 genomes analyzed according to the canSNP analysis. In this cluster, three strains (A0401, A3802 and A1144) coming from France, Ivory Coast, and Argentina were also included. Two Italian strains (A0293 and A0847) were part of the TEA sub-group 008/011, which was represented by 20 isolates from different countries throughout Europe and Asia.

The other two lineages B and C were represented by B.Br.001/002 and B.Br. Kruger (both established in Africa), B.Br.004 (France), and C. Br. A1055 and C. Br.001, which were found in USA.

### 3.3. Detailed Italian Genetic Positioning of the Abruzzo Outbreak Strain

**Figure 2:**
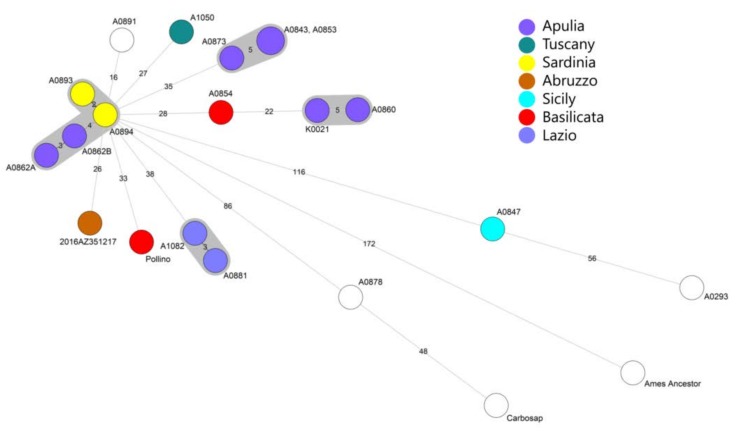
Minimum spanning tree of 20 Italian *B. anthracis* strains using SNP whole genome analysis. In the legend the regions to which the sequences belong are reported. The strains of unknown origin are represented with a white circle. The study strain is represented as a brown circle and as 2016AZ351217.

The results obtained from SNP whole genome analyses were used to build the minimum spanning tree (MST) for detailed phylogenetic analysis of the *B. anthracis* isolated in Abruzzo against the Italian isolates (Figure 2). Most of the *B. anthracis* strains from Italy belonged to the TEA sub-group A.Br.011/009.

The strain (2016.AZ.3512.1.7) from the outbreak was featured in the TEA sub-group A.Br.011/009. The MST discovered little genetic diversity among the strains from this study with the closest Sardinian strains (A0894 and A0893) and Apulian strains (A0862B and A0862A) that showed at least 28 SNPs of difference. The other Italian genomes from Apulia, Tuscany, Basilicata, and Lazio showed a higher number of SNPs from the Abruzzo outbreak.

For the strains A0891 and A0878, metadata were not available and the geographical origin was not possible to trace. The Carbosap strain was also excluded from the trace back analysis because it is a vaccine strain that was developed decades ago by Italian colleagues attenuating a wild African strain [33].

The A.Br.008/011, an early branch of TEA sub-group, was founded in Sicily (A0847) which was different from A.Br.011/009 with 116 SNPs, while there is no information about the geographical origin of the other strain (A0293). Overall, the clustering showed that the Italian isolates were split into five genetic clades, confirming the results by Sahl et al. [12].

**Figure 3:**
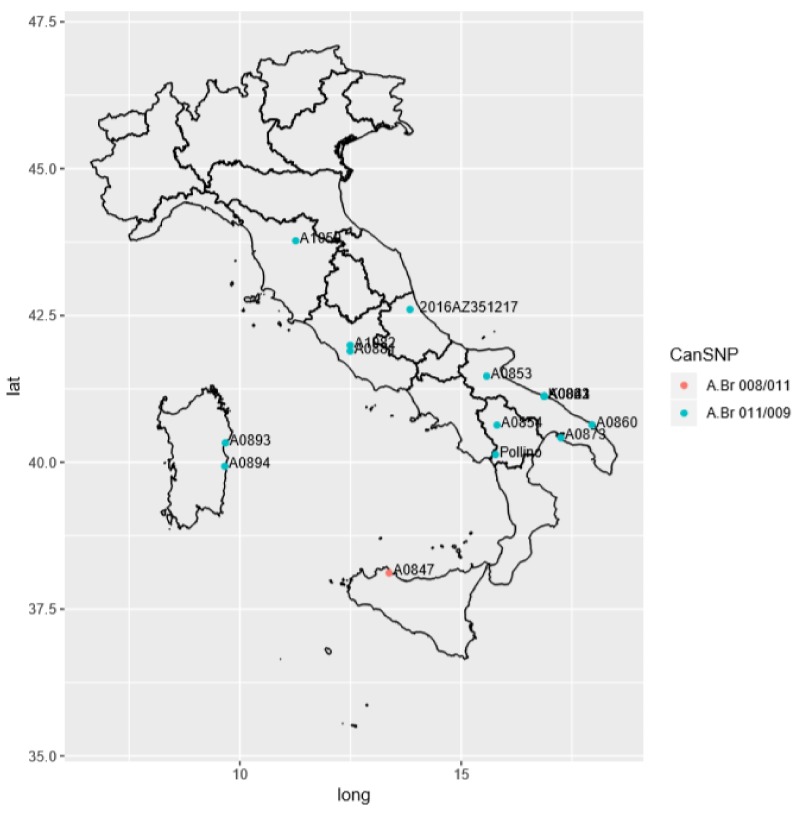
Geographical distribution of Italian strain representing one of the outputs of in-house pipeline used in this study. The legend reports the canSNP sub-group A.Br.008/011 and A.Br.011/009 as a red circle and blue circles, respectively. The study strain is represented as 2016AZ351217.

In summary, the distribution of Italian *B. anthracis* strains has shown to be linked to geographic areas; in fact, most isolates of *B. anthracis* are distributed in Central-Southern Italy, suggesting a common origin (Figure 3). Furthermore, the principle sources seem to be linked to human activities where the main hosts are represented by cattle, sheep, goats, and humans, as reported in Table S1.

## 4. Discussion and Conclusions

In this report, canSNP analysis and whole genome sequencing were used to investigate a *B. anthracis* outbreak in Italy for the first time.

The results from WGS analysis show a peculiar resistance profile of the study strain; indeed, the presence of resistance genes against vancomycin and macrolides has been found in the Abruzzo strain, although these molecules are usually effective in the therapy of *B. anthracis*. Meanwhile, the resistance to fosfomycin and the sensitivity to fluoroquinolones are confirmed as described previously by Schuch et al. [34] and Cavallo et al. [35].

The strain from the outbreak recorded in Abruzzo was compared with 19 isolates from Italy between the period 1972–2017, including Carbosap, a vaccine strain with residual pathogenicity, whose first use dates back to the 1940s [36].

The outbreak isolate was identified to belong to the most prevalent genetic sub-group in Italy, the TEA A.Br.009/011. The SNP analysis also found the TEA Br.008/011 in strains from the main island Sicily. Nonetheless, in this study, we did not find strains belonging to canSNP sub-groups B.Br. CNEVA and A.Br.005/006 that were previously found as less prevalent groups in Northeast Italy by Garofolo et al. [37].

Previous studies registered the TEA sub-group across Italy supporting the thesis of the introduction of one single ancestor in Italy in the past due to the close similarity of the *B. anthracis* strains [5]. Although the TEA sub-group is not exclusive in Italy, its widespread nature suggests rapid and extensive evolution in the Italian fields.

The regular droving of livestock that has taken place along the historical green ways known as “tratturi” might have helped to spread and maintain *B. anthracis* in the environment. The most recent common ancestor shared with Southern and Central Italian strains also strengthen the idea that anthrax spread through the movements of livestock along the green ways, and for this reason potentially new contaminated areas will be discovered.

Although classic methods of molecular characterization are used in most laboratories, public health facilities should implement the use of WGS for routine surveillance and outbreak studies [38].

This study demonstrated the use of WGS as potential tool for supporting the epidemiological analysis of an outbreak and surveillance activities.

The interpretation of data deriving from the combination of epidemiological data and the characterization of *B. anthracis* genomes present in the territory could guide public health action in an outbreak scenario. To this end, it is necessary to obtain a greater number of strains sequenced from the country, which reflect the wider genetic and geographical diversity; in this way, it would be possible to obtain a detailed picture of the epidemiological situation of anthrax. Indeed, the scarcity of Italian sequences present in Genbank could limit the trace back operations in the event of an outbreak, highlighted as a precedent by the lack of isolates belonging to the canSNP sub-groups CNEVA and A.Br.005/006, described by Garofolo et al. [37].

Furthermore, our study can contribute to the construction of an analytical workflow involving WGS data and epidemiological interpretation criteria that can be used by anthrax national reference laboratories around the world. Thus, this work represents a preliminary stage for a future multi-level assessment of the effectiveness of public health of WGS-based typing in surveillance programs and in the analysis of future outbreaks. In the future, through the use of the information suggested by the WGS data, it will be possible to map the areas at risk for a possible spread of the disease.

## Figures and Tables

**Table 1:** Genomic feature of outbreak strain.

**Parameter**	**Characteristics of the Outbreak Strain**
Total number of reads	4,564,206
Total number of nucleotides	615,149,873
G + C content	36.0
Average read length	134 (35–151)
Depth of coverage	109x
**Genome assembly results**	
Total number of contigs	35
Total genome length	5,450,929 bp
Average contig size	155,740 bp (524–1,052,202 bp)
N50	514,228 bp
**Genome annotation results**	
Total number of CDS	5718
Total number of rRNA	7
Total number of tRNA	80
Total number of tmRNA	1
**Virulence genes**	*nheA*; non-hemolytic enterotoxin A—NP_978284
	*nhe*; non-hemolytic enterotoxin B—NP_978285
	*nhe*; non-hemolytic enterotoxin C—NP_978286
	*BAS3109*; thiol-activated cytolysin—YP_029366
	*inhA*; immune inhibitor A metalloprotease—YP_026915
	*capE*; CapE involved in Poly-gamma-glutamate synthesis—YP_016572
	*dep/capD*; gamma-glutamyltranspeptidase required for polyglutamate anchoring to peptidoglycan—AAF13660
	*capA*; CapA required for Poly-gamma-glutamate transport—AAF13661
	*capC*; CapC involved in Poly-gamma-glutamate synthesis—AAF13662
	*capB*; CapB involved in Poly-gamma-glutamate synthesis—AAF13663
	*cya*; calmodulin sensitive adenylate cyclase edema factor—AAD32426
	*pagA*; anthrax toxin moiety protective antigen—AAD32414
	*lef*; anthrax toxin lethal factor precursor—AAD32411
**Resistance genes**	*fosB*; FosB/FosD family fosfomycin resistance bacillithiol transferase—A7J11_05167
	*bla*; class A beta-lactamase Bla1—A7J11_05168
	*lsa(B)*; ABC-F type ribosomal protection protein Lsa(B)—A7J11_01190
	*fosB2*; fosfomycin resistance bacillithiol transferase FosB2—A7J11_00634
	*bla2*; BcII family subclass B1 metallo-beta-lactamase—A7J11_00039
	*mph(B)*; Mph(B) family macrolide 2’-phosphotransferase—A7J11_05208
	*vanZ-F*; glycopeptide resistance protein VanZ-F—A7J11_00543
	*vanR*; VanM-type vancomycin resistance DNA-binding response regulator VanR—A7J11_02292

## Supplementary Materials

The following are available online at https://www.mdpi.com/2076-2607/8/1/87/s1. Table S1: Sequencing IDs, epidemiological information and references of all 91 *B. anthracis* strains used in this study. Table S2: Plasmids detected in Italian *B. anthracis* strains
