# Supplementary material for: Whole Genome Sequencing for Studying Bacillus anthracis from an Outbreak in the Abruzzo Region of Italy
Source: Microorganisms. 2020 Jan 8;8(1):87. doi: 10.3390/microorganisms8010087 (PMC7022239; doi:10.3390/microorganisms8010087)
Supplement: Supplementary file 1 [file microorganisms-08-00087-s001.zip › Supplement materials/Table S1_Sequencing IDs, epidemiological information and references of all 91 B. anthracis strains used in this study_corrected.pdf]

**Table S1.** Sequencing IDs, epidemiological information and references of all 91 *B. anthracis* strains used in this study

| ID | Country | Source | Year | SNP_group | Accession number | Reference |
|---|---|---|---|---|---|---|
| **2016.AZ.3512.1.7** | Italy | Goat | 2016 | A_Br_011_009 | SRR10402317 | This study |
| **A0264** | Turkey | Human | 1994 | A_Br_008_011 | SRR19994417 | [12] |
| **A0088** | South_Africa | / | / | A_Br_002_003 | SRR2968133 | [12] |
| **A0091** | South_Africa | / | 1939 | B_Br_001_002 | SRR2968134 | [12] |
| **A0094** | South_Africa | / | 1949 | A_Br_003_004 | SRR2968135 | [12] |
| **A0293** | Italy | Sheep | / | A_Br_008_011 | SRR2968137 | [12] |
| **A0684** | China | Soil | 1982 | A_Br_008_011 | SRR2968138 | [12] |
| **A1035** | Albania | Sheep | / | A_Br_008_011 | SRR2968140 | [12] |
| **A0002** | Turkey | / | / | A_Br_002_003 | SRR2968144 | [12] |
| **A0656** | China | Soil | 1982 | A_Br_002_003 | SRR2968145 | [12] |
| **A1096** | Argentina | Bovine | / | A_Br_003_004 | SRR2968148 | [12] |
| **A3716** | Namibia | Zebra | 2006 | A_Br_002_003 | SRR2968149 | [12] |
| **A0006** | Australia | / | / | A_Br_005_006 | SRR2968150 | [12] |
| **A0017** | Zambia | / | / | A_Br_005_006 | SRR2968151 | [12] |
| **A0026** | UK | Equine | 1992 | A_Br_005_006 | SRR2968152 | [12] |
| **A0062** | Poland | / | / | A_Br_008_011 | SRR2968154 | [12] |
| **A0083** | Germany | Soil | / | A_Br_002_003 | SRR2968155 | [12] |
| **A0224** | Australia | Flies | / | A_Br_002_003 | SRR2968158 | [12] |
| **A0245** | Turkey | Bovine | / | A_Br_008_011 | SRR2968159 | [12] |
| **A0252** | Zimbabwe | / | / | A_Br_002_003 | SRR2968160 | [12] |
| **A0363** | Norway | Bovine | / | A_Br_007 | SRR2968161 | [12] |
| **A0380** | UK | wool | / | A_Br_007 | SRR2968162 | [12] |
| **A0386** | UK | Human | / | A_Br_002_003 | SRR2968163 | [12] |
| **A0404** | India | Human | / | A_Br_002_003 | SRR2968164 | [12] |
| **A0455** | Mozambique | Bovine | 1965 | A_Br_002_003 | SRR2968165 | [12] |
| **A0464** | Indonesia | Bovine | / | A_Br_005_008_005_007 | SRR2968166 | [12] |
| **A0478** | Spain | Environment | / | A_Br_007 | SRR2968167 | [12] |
| **A0481** | Russia | Vaccine | / | A_Br_008_011 | SRR2968168 | [12] |
| **A0530** | Botwana | elephant | / | A_Br_005_006 | SRR2968170 | [12] |
| **A0533** | South_Africa | Goat | / | A_Br_005_006 | SRR2968171 | [12] |
| **A0615** | China | Soil | 1981 | A_Br_007 | SRR2968173 | [12] |
| **A0843** | Italy | Sheep | 1993 | A_Br_011_009 | SRR2968174 | [12] |
| **A0847** | Italy | Sheep | 1997 | A_Br_008_011 | SRR2968175 | [12] |
| **A0853** | Italy | Bovine | 1999 | A_Br_011_009 | SRR2968176 | [12] |
| **A0854** | Italy | Bovine | 1999 | A_Br_011_009 | SRR2968177 | [12] |
| **A0860** | Italy | Sheep | 1984 | A_Br_011_009 | SRR2968178 | [12] |

| | | | | | | |
|---|---|---|---|---|---|---|
| **A0862A** | Italy | Sheep | 1984 | A_Br_011_009 | SRR2968179 | [12] |
| **A0873** | Italy | Bovine | 1972 | A_Br_011_009 | SRR2968180 | [12] |
| **A0878** | Italy | / | / | A_Br_011_009 | SRR2968181 | [12] |
| **A0881** | Italy | Human | / | A_Br_011_009 | SRR2968182 | [12] |
| **A1058** | USA | Bovine | 1957 | A_Br_007 | SRR2968183 | [12] |
| **A1063** | USA | / | 1960 | A_Br_007 | SRR2968184 | [12] |
| **A1082** | Italy | Bovine | / | A_Br_011_009 | SRR2968185 | [12] |
| **A2075** | Tanzania | Baboon | 1999 | A_Br_005_006 | SRR2968187 | [12] |
| **A2079** | Tanzania | Impala | 1999 | A_Br_005_006 | SRR2968188 | [12] |
| **A0001** | Turkey | / | / | A_Br_002_003 | SRR2968189 | [12] |
| **A0081** | Germany | Soil | 1998 | A_Br_003_004 | SRR2968190 | [12] |
| **A0096** | South_Africa | / | 1939 | A_Br_003_004 | SRR2968191 | [12] |
| **A0097** | South_Africa | / | 1938 | A_Br_003_004 | SRR2968192 | [12] |
| **A0103** | Turkey | Bovine | / | A_Br_002_003 | SRR2968193 | [12] |
| **A0148** | Turkey | Human | 1990 | A_Br_002_003 | SRR2968194 | [12] |
| **A1101** | Argentina | Bovine | / | A_Br_003_004 | SRR2968195 | [12] |
| **A1102** | Argentina | Pig | / | A_Br_003_004 | SRR2968196 | [12] |
| **A11193** | Bulgaria | Bovine | 1960_80 | A_Br_008_011 | SRR2968197 | [12] |
| **A0150** | Turkey | Human | 1995 | A_Br_008_011 | SRR2968200 | [12] |
| **A0303** | Canada | Bovine | / | A_Br_009 | SRR2968201 | [12] |
| **A0362** | Norway | Bovine | / | A_Br_008_011 | SRR2968203 | [12] |
| **A0401** | France | Bovine | / | A_Br_011_009 | SRR2968204 | [12] |
| **A0417** | Hungary | / | / | A_Br_008_011 | SRR2968172 | [12] |
| **A0675** | China | Soil | 1982 | A_Br_008_011 | SRR2968207 | [12] |
| **A0862B** | Italy | Sheep | 1984 | A_Br_011_009 | SRR2968209 | [12] |
| **A0891** | Italy | Goat | 1989 | A_Br_011_009 | SRR2968210 | [12] |
| **A0893** | Italy | Goat | 1996 | A_Br_011_009 | SRR2968211 | [12] |
| **A0894** | Italy | Goat | 1997 | A_Br_011_009 | SRR2968212 | [12] |
| **A1050** | Italy | Bovine | / | A_Br_011_009 | SRR2968214 | [12] |
| **A3802** | Ivory coast | Goat hide | 2006 | A_Br_011_009 | SRR2968215 | [12] |
| **Shikan_NID** | Japan | Equine | 1928 | A_Br_001 | GCA_002356575.1_ASM235657v1 | [12] |
| **RA3** | France | Bovine | 1997 | B_Br_004 | GCA_000832745.1_ASM83274v1 | [12] |
| **A1144** | Argentina | Bovine | / | A_Br_011_009 | GCA_000875715.1_ASM87571v1 | [12] |
| **A1045** | USA | Bovine | 1952 | A_Br_001_002 | GCA_000008165.1_ASM816v1 | [12] |
| **Ames_ancestor** | USA | Bovine | | A_Br_Ames | GCA_000008445.1_ASM844v1 | [12] |
| **BFV** | Jamaica | Environment | / | A_Br_001_002 | GCA_000742875.1_ASM74287v1 | [12] |
| **K0021** | Italy | Sheep | / | A_Br_011_009 | GCA_001273045.1_BANT008-SEQ-1-ASM-1 | [12] |
| **A0937** | Thailand | / | / | A_Br_001_002 | GCF_000007845.1_ASM784v1 | [12] |

| | | | | | | |
|---|---|---|---|---|---|---|
| **A1055** | USA | Soil | 1956 | C_Br_A1055 | GCA_000167255.1_ASM16725v1 | [12] |
| **PAK-1** | Pakistan | Sheep | 1978 | A_Br_008_011 | GCF_000832425.1_ASM83242v1 | [12] |
| **Canadian_bison** | USA | Bison | / | A_Br_009 | GCA_000833125.1_ASM83312v1 | [12] |
| **Pasteur** | UK | Cat | / | A_Br_008_011 | GCA_000832585.1_ASM83258v1 | [12] |
| **BA1015** | USA | Bovine | 1939 | A_Br_003_004 | GCA_000832665.1_ASM83266v1 | [12] |
| **2002013094** | USA | Environment | 1956 | C_Br_001 | GCA_000783235.1_ASM78323v1 | [12] |
| **Australia94** | Australia | Bovine | 1994 | A_Br_Aust94 | GCA_000167235.1_ASM16723v1 | [12] |
| **Carbosap** | Italy | / | 1940_45 | A_Br_011_009 | GCF_000310045.1_Carbosap_v1 | [12] |
| **3154** | Bulgaria | Soil | 1960_80 | A_Br_008_011 | GCA_000319695.1_BA_3154assembly | [12] |
| **Kruger_B** | South_Africa | / | / | B_Br_KrugerB | GCA_000167295.1_ASM16729v1 | [12] |
| **Tsiankovskii_I** | Russia | Vaccine | 1960 | A_Br_008_011 | GCF_000181675.2_ASM18167v2 | [12] |
| **Pollino** | Italy | Bovine | / | A_Br_011_009 | GCA_000831505.1_ASM83150v1 | [12] |
| **A4566** | Denmark | Human | 2012 | A_Br_008_011 | SRR1999716 | [12] |
| **A4568** | Norway | Human | 2000 | A_Br_008_011 | SRR1999718 | [12] |
| **A0696** | China | soil | 1982 | A_Br_008_011 | SRR2968208 | [12] |
| **A0033** | China | wool | / | A_Br_008_011 | SRR2968153 | [12] |
| **K2129** | China | fur | / | A_Br_008_011 | LFYG01000000.1 | [12] |
