# Supplementary material for: Whole Genome Sequencing for Studying Bacillus anthracis from an Outbreak in the Abruzzo Region of Italy
Source: Microorganisms. 2020 Jan 8;8(1):87. doi: 10.3390/microorganisms8010087 (PMC7022239; doi:10.3390/microorganisms8010087)
Supplement: Supplementary file 1 [file microorganisms-08-00087-s001.zip › Supplement materials/Table S2_Plasmids detected in Italian B. anthracis strains_revised.pdf]

Table S2: Plasmids detected in Italian *B. anthracis* strains

ID	pOX1_cya	pOX1_lef	pOX1_pag	pOX1_repX	pOX2_capA	pOX2_capB	pOX2_capC	pOX2_capD	pOX2_capE	pOX2_repS	pOX1	pOX2
2016.AZ.3512.1.7	100.00	100.00	100.00	100.00	100.00	100.00	100.00	100.00	100.00	100.00	present	present
A0293	100.00	100.00	100.00	100.00	100.00	100.00	100.00	100.00	100.00	100.00	present	present
A0843	100.00	100.00	100.00	100.00	100.00	100.00	100.00	100.00	100.00	100.00	present	present
A0847	100.00	100.00	100.00	100.00	100.00	100.00	100.00	100.00	100.00	100.00	present	present
A0853	100.00	100.00	100.00	100.00	100.00	100.00	100.00	100.00	100.00	100.00	present	present
A0854	100.00	100.00	100.00	100.00	100.00	100.00	100.00	100.00	100.00	100.00	present	present
A0860	100.00	100.00	100.00	100.00	100.00	100.00	100.00	100.00	100.00	100.00	present	present
A0862A	100.00	100.00	100.00	100.00	100.00	100.00	100.00	100.00	100.00	100.00	present	present
A0862B	100.00	49.55;53.21	100.00	100.00	100.00	100.00	100.00	100.00	100.00	100.00	present	present
A0873	100.00	100.00	100.00	100.00	100.00	100.00	100.00	100.00	100.00	100.00	present	present
A0878	0,00	0,00	0,00	0,00	0,00	0,00	0,00	0,00	0,00	0,00	absent	absent
A0881	100.00	100.00	100.00	100.00	100.00	100.00	100.00	100.00	100.00	100.00	present	present
A0891	100.00	100.00	100.00	100.00	100.00	100.00	100.00	100.00	100.00	100.00	present	present
A0893	100.00	100.00	100.00	100.00	100.00	100.00	100.00	100.00	100.00	100.00	present	present
A0894	100.00	100.00	100.00	100.00	100.00	100.00	100.00	100.00	100.00	100.00	present	present
A1050	100.00	100.00	100.00	100.00	100.00	100.00	100.00	100.00	100.00	100.00	present	present
Ames_ancestor	100.00	100.00	100.00	100.00	100.00	100.00	100.00	100.00	100.00	100.00	present	present
Carbosap	100.00	100.00	100.00	100.00	100.00	100.00	100.00	100.00	100.00	100.00	present	present
K0021	100.00	100.00	100.00	100.00	100.00	100.00	100.00	100.00	100.00	100.00	present	present
Pollino	100.00	100.00	100.00	100.00	100.00	100.00	100.00	100.00	100.00	100.00	present	present
